# Clinical and Economic Burden of Invasive Pneumococcal Disease in Children in Suriname: A Nationwide Cohort Study From a Middle-income Country in the Absence of a Pneumococcal Immunization Program

**DOI:** 10.1097/INF.0000000000005183

**Published:** 2026-02-18

**Authors:** Kevin H. van ’t Kruys, Frederick W. Thielen, Nikita Borstlap, Terrence Mawie, Gertjan J. Driessen, Frans B. Plötz, Amadu E. Juliana, Navin P. Boeddha

**Affiliations:** From the * Department of Pediatrics, Academic Hospital Paramaribo, Paramaribo, Suriname; † Erasmus School of Health Policy and Management, Erasmus University Rotterdam, Rotterdam, The Netherlands; ‡ Erasmus Centre for Health Economics Rotterdam, Erasmus University, Rotterdam, The Netherlands; § Faculty of Medicine, Amsterdam UMC, location Vrije Universiteit Amsterdam, Amsterdam, The Netherlands; ¶ Department of Medical Microbiology, Academic Hospital Paramaribo, Paramaribo, Suriname; ‖ Department of Pediatrics, MosaKids Children’s Hospital, Maastricht University Medical Centre, Maastricht, The Netherlands; ** Department of Pediatrics, Tergooi Hospital, Hilversum, The Netherlands; †† Department of Pediatrics and Amsterdam Reproduction & Development Research Institute, Emma Children’s Hospital, Amsterdam UMC, location University of Amsterdam, Amsterdam, The Netherlands; ‡‡ Department of Pediatrics, Maasstad Hospital, Rotterdam, The Netherlands; §§ Division of Pediatric Infectious Diseases and Immunology, Department of Pediatrics, Erasmus MC-Sophia Children’s Hospital, University Medical Centre Rotterdam, Rotterdam, The Netherlands.

**Keywords:** cost of illness, incidence, pneumococcal infections, resource-limited settings, vaccination

## Abstract

This nationwide cohort study (2017 to 2022) from Suriname, one of the 25 countries worldwide lacking pneumococcal conjugate vaccines in its national immunization program, reveals a significant clinical burden of invasive pneumococcal disease, with pneumonia being the most common manifestation. High mortality, frequent disability and substantial healthcare costs, especially for children admitted to the pediatric intensive care unit, advocate for the introduction of a pneumococcal immunization program.

*Streptococcus pneumoniae* can progress from nasopharyngeal colonization to invasive pneumococcal disease (IPD), such as pneumonia or sepsis. Before the introduction of pneumococcal conjugate vaccines (PCV), the World Health Organization estimated that 1 million children died annually from IPD, accounting for 8%–12% of all deaths among children under 5.^1^ Apart from mortality, IPD causes significant disability such as motor deficits, seizures or hearing loss, and high costs for households and healthcare systems. Consequently, the World Health Organization recommends routine PCV administration for all countries.

PCV are now widely available, have significantly reduced IPD cases, mortality and morbidity, and are cost-effective in both high-income and low-and-middle-income countries.^2^

Suriname, a middle-income country in South America with 600,000 inhabitants, has not yet implemented PCV in its national immunization program.^3^ Because a national surveillance system is lacking, data on the clinical burden and IPD-related costs do not exist. This study aimed to assess the incidence, clinical characteristics, mortality, disability and risk factors for adverse outcome of children with IPD in Suriname. Additionally, we studied costs associated with episodes of IPD.

## MATERIALS AND METHODS

This nationwide, retrospective cohort study involved all 5 hospitals providing pediatric care in Suriname. The main study center, Academic Hospital Paramaribo (AHP), is the only university hospital including a pediatric intensive care unit (PICU). Healthcare in the inland of Suriname is provided at small primary healthcare facilities. Body materials from all health facilities in the country are sent to the Department of Medical Microbiology at AHP for microbiologic diagnostics.

From the culture registry at AHP, we identified and collected data from all children (0–18 years) with IPD between January 1, 2017, and August 31, 2022. We defined IPD as the detection (by culture) of *S. pneumoniae* in a normally sterile site (ie, blood, cerebrospinal fluid, bronchoalveolar lavage, joint aspirate, abscess aspirate, intraoperative swabs and pleural aspirate). Annual incidence rate per 100,000 children was calculated by dividing yearly positive cultures by relevant population (children 0–15 years or those younger than 5 years).^4^ From the data available, we examined 4 risk factors for adverse outcome: age, underlying condition, underweight and clinical manifestation. Underlying conditions followed the pediatric complex chronic conditions system. Underweight was defined as a weight-for-age Z score below −2 standard deviation (SD). For laboratory tests, we included the first result within 24 hours of presentation to the hospital. Disability was defined as any sequelae (ie, any complication or condition) recorded at the final follow-up visit. Serotype data were unavailable.

We also analyzed hospital bills from AHP to estimate the economic burden, focusing on inpatient costs. Cost components included consumables, medication, laboratory examinations, imaging, specialist consultations and hospitalization. Costs of patients with and without PICU visit were compared. Costs were converted from Surinamese Dollars (SRD) to 2022 US and International Dollars (US$, I$). See Tables, Supplemental Digital Content 1–3, https://links.lww.com/INF/G564 and Figure, Supplemental Digital Content 4, https://links.lww.com/INF/G564 for more details.

Statistical analysis and ethical considerations are presented in Table, Supplemental Digital Content 5 https://links.lww.com/INF/G564.

## RESULTS

We included 46 patients (52% male, median age 11 months [interquartile range (IQR) 5–28]), with *S. pneumoniae* isolated from 54 sterile body sites (Table 1). The mean annual incidence rate was 5.5 (95% confidence interval: 4.0–7.0) per 100,000 children 0–15 years, with a higher rate of 14.2 (95% confidence interval: 10.1–18.3) per 100,000 for children under 5. Incidence in children under 5 ranged from 5.5 to 24.5 per 100,000 throughout the study period (Table, Supplemental Digital Content 6, https://links.lww.com/INF/G564).

**TABLE 1.** Characteristics of Children Admitted With IPD

	All Patients (n = 46)	No PICU Admission^a^ (n = 27)	PICU admission^a^ (n = 18)	*P*
Sex (male n, %)	24 (52%)	15 (56%)	9 (50%)	ns
Age (months)	11 [5-28]	16 [7-30]	6 [2-19]	ns
Age group				ns
<2 yr	32 (70%)	17 (63%)	14 (78%)	
2–5 yr	10 (22%)	8 (30%)	2 (11%)	
>5 yr	4 (9%)	2 (7%)	2 (11%)	
Hospital of positive culture				ns
Academic Hospital Paramaribo	31 (67%)	15 (56%)	16 (89%)	
Diakonessen Hospital	7 (15%)	6 (22%)	1 (6%)	
‘s Lands Hospital	4 (9%)	3 (11%)	0 (0%)	
Sint Vincentius Hospital	4 (9%)	3 (11%)	1 (6%)	
Mungra Medical Centre	0 (0%)	0 (0%)	0 (0%)	
Underlying condition (yes n, %)	17 (37%)	6 (22%)	10 (59%)	0.01
Underweight^b^ (yes n, %)	8 (19%)	4 (16%)	4 (22%)	ns
Time between onset of symptoms and date of culture^c^ (d)	3 [2−3]	3 [2−4]	2 [2−3]	ns
Clinical manifestation				0.04
Pneumonia	19 (41%)	14 (52%)	5 (28%)	
Meningitis	12 (26%)	4 (15%)	8 (44%)	
Bacteremia/sepsis	11 (24%)	5 (19%)	5 (28%)	
Other	4 (9%)	4 (15%)	−	
Site of infection^d^				ns
Blood	39 (85%)	23 (85%)	15 (83%)	
Cerebrospinal fluid	9 (20%)	3 (11%)	6 (33%)	
Pleural aspirate	1 (2%)	0 (0%)	1 (6%)	
Abscess aspirate	4 (9%)	3 (11%)	1 (6%)	
Joint aspirate	1 (2%)	1 (4%)	0 (0%)	
Laboratory tests				
First platelets^e^ (×10e9/L)	402 [268−539]	405 [296−527]	335 [217−619]	ns
First leukocytes^f^ (×10e9/L)	25 [18.4−34.4]	26.6 [20.2−36.6]	19.9 [8.7−30.8]	ns
First CRP^g^ (mg/L)	220 [123−316]	211 [108−262]	236 [140−359]	ns
Maximum CRP^h^ (mg/L)	243 [137−351]	211 [126−262]	334 [234−395]	ns
First lactate^i^ (mmol/L)	5.0 [2.1−13.4]	1.7 [0.9–−]	8.4 [4.4−16]	0.02
Outcome				
Hospital length of stay^j^ (days)	9 [4−17]	6 [4−13]	16 [4−30]	0.03
Mortality (yes n, %)	7 (15%)	1 (4%)	6 (33%)	<0.01
Disability^k^ (yes n, %)	8 (40%)	7 (44%)	5 (63%)	0.04

Values are reported as counts (percentages) or medians [interquartile ranges], unless stated otherwise.

a PICU admission was unknown for 1 patient.

b Weight was known for 43/46 patients: 25/27 non-PICU and 18/18 PICU patients.

c Time between onset of symptoms and date of culture was available for 29/46 patients: 20/27 non-PICU and 9/18 PICU patients.

d Total count exceeds 46 because some patients had positive cultures from multiple sites.

e First platelets data was available for 34/46 patients: 21/27 non-PICU and 13/18 PICU patients.

f First leukocytes data was available for 35/46 patients: 22/27 non-PICU and 13/18 PICU patients.

g First CRP data was available for 33/46 patients: 19/27 non-PICU and 14/18 PICU patients.

h Maximum CRP data were available for 31/46 patients: 19/27 non-PICU and 12/18 PICU patients.

i First lactate data was available for 10/46 patients: 3/27 non-PICU and 7/18 PICU patients.

j Hospital length of stay data was available for 44/46 patients: 27/27 non-PICU and 17/18 PICU patients.

k Disability data was available for 20/39 survivors: 16/27 non-PICU and 8/12 PICU survivors.

CRP indicates C-reactive protein.

An underlying condition was present in 17/46 (37%) of the children. The most common conditions were cardiovascular (3/17, 18%), hematologic or immunologic (3/17, 18%), and prematurity or neonatal conditions (3/17, 18%). Additionally, 8/43 (19%, 3 missing data) were underweight. The median time between onset of symptoms and culture was 3 days (IQR 2–3). None of the patients were vaccinated against *S. pneumoniae*. The most common clinical manifestations were pneumonia (n = 19, 41%), meningitis (n = 12, 26%) and bacteremia/sepsis (n = 11, 24%). The median hospital stay was 9 days (IQR 4–17).

Eighteen of 45 children (40%) were admitted to the PICU (50% male, median age 6 months (IQR 2–19), 59% with an underlying condition), with meningitis (n = 8, 44%) being the most common clinical manifestation. Twelve of 15 (75%) PICU patients required mechanical ventilation (median 7 days, IQR 3–8) and 3/14 (21%) received inotropes (median 5 days, IQR 1–21). The median PICU stay was 13 days (IQR 4–22). Children admitted to the PICU more frequently had underlying conditions, higher lactate levels at presentation and longer hospital stays compared to children not admitted to the PICU (*P* < 0.05).

Mortality occurred in 7/46 (15%) children, most of whom suffered from bacteremia/sepsis (n = 3, 43%). Data on disability was available for 20/39 (51%) survivors. The remaining survivors were lost to follow-up. Eight of 20 (40%) children had some form of disability. Sequelae were neurologic, including seizures (n = 7), permanent brain damage with cognitive impairment and/or motor dysfunction (n = 3) and hearing loss (n = 2). Analysis of potential risk factors for adverse outcome showed that diagnosis of meningitis was independently associated (*P* < 0.05) with mortality and/or disability (Table, Supplemental Digital Content 7, https://links.lww.com/INF/G564).

Hospital bills were available for 19/46 (41%) children, 12 of whom had a PICU admission. Mean total costs per patient were US$ 1014.15 (SD ±906.13), ranging from US$ 45.98 to US$ 2627.82. Hospitalization was the largest component (US$ 486.83, SD ±476.26), followed by medication (US$ 249.59, SD ±340.65), while imaging costs (US$ 30.37, SD ±25.13) were lowest (Table, Supplemental Digital Content 8, https://links.lww.com/INF/G564). Cost expressed in I$ are presented in Table, Supplemental Digital Content 9, https://links.lww.com/INF/G564. Because bills came from the only hospital with a PICU, PICU admission was more common in this subgroup (Table, Supplemental Digital Content 10, https://links.lww.com/INF/G564). Mean costs for children with PICU admission (US$ 1452.13, SD ±883.79) were higher compared to those without PICU admission (US$ 309.63, SD ±338.09) (Table, Supplemental Digital Content 8, https://links.lww.com/INF/G564).

## DISCUSSION

This nationwide cohort study from Suriname provides an unique and contemporary insight into IPD in one of the 25 countries worldwide lacking PCV in its national immunization program.^3^ Our study reveals a significant clinical burden, with pneumonia being the most common manifestation. Mortality was 15% and 40% of survivors had a form of disability. Additionally, we identified substantial costs, especially for children admitted to the PICU.

In children under 5, the mean annual incidence was 14.2/100,000 children, which is higher than in other middle-income countries in Latin America in the pre-PCV-era (eg, 2.9/100,000 in Costa Rica and 8.0/100,000 in Peru).^5,6^ Incidence varied throughout the study period, with nadir in 2020 and peak in 2022, possibly related to measures to prevent transmission in the coronavirus disease 2019 era.^7^

We probably underestimate the true incidence of IPD in Suriname for several reasons. Confirming *S. pneumoniae* as the cause of pneumonia is challenging. Adult studies estimate that for every case of bacteremic pneumococcal pneumonia, at least 3 additional cases of non-bacteremic pneumonia go undiagnosed, thereby highlighting the potential underestimation of IPD in Suriname.^8^ Additionally, pneumococcal PCR for culture-negative meningitis is not available in Suriname. Lastly, logistic challenges may have hindered the timely collection and transportation of sterile site samples for culture, potentially resulting in the underdiagnosis of IPD.

The mortality rate of 15% in our cohort is comparable to pre-PCV rates in surrounding countries (Costa Rica 14%,^5^ Peru 22%^6^). In addition to mortality, 40% of IPD survivors had disability, consistent with outcomes of children with sepsis, where *S. pneumoniae* infection is an independent predictor for adverse outcome.^9^ Meningitis was associated with mortality and/or disability, as previously reported.^5^

We found considerable variations in costs, with hospitalization as largest cost driver, followed by medication. However, it is important to note that the costs per patient only represent healthcare expenses and underestimate the actual costs, as they omit societal costs like travel expenses or caregiver productivity losses. Direct comparisons with other cost-of-illness studies are difficult due to differences in healthcare systems, methodologies and economic contexts.

This study has several limitations. First, the cohort is relatively small, but given the nationwide design and the presence of only one microbiology laboratory, we believe it provides valuable data. Second, disability data were unknown for almost half of survivors, hindering more accurate analysis of outcome. Future studies should emphasize on follow-up and specify long-term disabilities to better assess the impact of IPD on children’s health in Suriname. Lastly, we lack data for a comprehensive cost-effectiveness analysis, for example, serotype data, vaccine procurement costs, vaccine delivery systems and long-term data on vaccine effectiveness. However, evidence strongly supports that infant PCV programs are cost-effective.^2^ Despite these limitations, our study provides valuable insights into the clinical and economic burden of IPD in Suriname, which could inform decisions regarding pneumococcal immunization. We report comparable pre-PCV incidence and outcomes as countries that have benefited from PCV vaccination regarding incidence, hospitalization, and outcome.^10^ The next step for Suriname could be to implement a prospective surveillance program, including the availability of PCR technique and serotyping of *S. pneumoniae*.

In conclusion, this study highlights the significant clinical and economic burden of IPD in the absence of an immunization program. The high incidence, especially in children under 5, and substantial healthcare costs, particularly for PICU admissions, advocates for the introduction of a pneumococcal conjugate immunization program.

## Acknowledgments

*The authors acknowledge all pediatricians from the five hospitals providing healthcare to children in Suriname for their support. We are also grateful to the personnel of the Department of Medical Microbiology and the medical archives at the Academic Hospital Paramaribo, and to medical student Janice Ferdinand for their valuable assistance with data collection. We used ChatGPT to correct language errors and to improve the readability of our manuscript.*

## Supplementary Material


