# Supplementary material for: Clinical and Economic Burden of Invasive Pneumococcal Disease in Children in Suriname: A Nationwide Cohort Study From a Middle-income Country in the Absence of a Pneumococcal Immunization Program
Source: Pediatr Infect Dis J. 2026 Feb 18;45(9):812–5. doi: 10.1097/INF.0000000000005183 (PMC13446908; doi:10.1097/INF.0000000000005183)
Supplement: Supplementary file 1 [file inf-45-812-s001.pdf]

SUPPLEMENTAL DIGITAL CONTENT 1. Cost conversion

Recognising the importance of international comparability of our findings, we converted costs stated in Surinamese Dollars (SRD) to 2022 US Dollars (US$), and International Dollars (I$) using purchasing power parities (PPP). To this end, we followed the methodology outlined by Turner et al.[1] and grouped all cost into tradeable and non-tradeable goods before conversion (see Figure, Supplemental Digital Content 4). Tradeable goods were first converted to US$ / I$ using the exchange rate at the time of costing and subsequently inflated using US$ inflation rates using the US consumer price index (CPI). Non-tradeable goods were first inflated using Surinamese consumer price index (CPI) rates and then converted to US$ / I$. Values for the PPP and CPI were taken from published data of the World Bank via the R package "wbstats" (Version 1.0.4), and historical exchange rates were taken from the R package "priceR" (Version 1.01.). Of note, the CPI index for 2018 was missing, which seems to be a common problem in Suriname[2]. We therefore used the CPI index for 2019 for all costs incurred in 2018 (i.e., last observation carried forward as recommended by the International Monetary Fund) [2]. Conversion factors and CPIs used for this analysis are summarized in Tables, Supplemental Digital Content 2 and 3. Given that most studies in this field provide estimates in US$, we follow this approach in this article and report I$ in Table, Supplemental Digital Content 9.

REFERENCES

1. Turner HC, Lauer JA, Tran BX, Teerawattananon Y, Jit M. Adjusting for Inflation and Currency Changes Within Health Economic Studies. Value Health. 2019;22(9):1026-32.
2. Suriname: Technical Assistance Report - CARTAC Report on the Price Statistics Mission. IMF Country Report No. 18/201. : International Monetary Fund. Statistics Dept.; 2018 [Available from: https://www.elibrary.imf.org/view/journals/002/2018/201/article-A001-en.xml.

SUPPLEMENTAL DIGITAL CONTENT 2. Conversion factors based on World Bank data through *priceR* (US$) and *wbstats* (I$) R-packages

**From SRD to**	**Year**					
	**2017**	**2018**	**2019**	**2020**	**2021**	**2022**
US$	0.135	0.135	0.134	0.117	0.057	0.042
IS$	0.387	0.375	0.363	0.253	0.165	0.122

SUPPLEMENTAL DIGITAL CONTENT 3. Consumer prices indexes based on World Bank data through the *wbstats* R-package

**Country**	**Year**					
	**2017**	**2018**	**2019**	**2020**	**2021**	**2022**
USA	112.41	115.15	117.24	118.69	124.26	134.21
Suriname	263.96	263.96	294.68	397.49	632.49	964.21

SUPPLEMENTAL DIGITAL CONTENT 4. Application of the Turner method for tradeable (A) and non-tradeable (B) goods

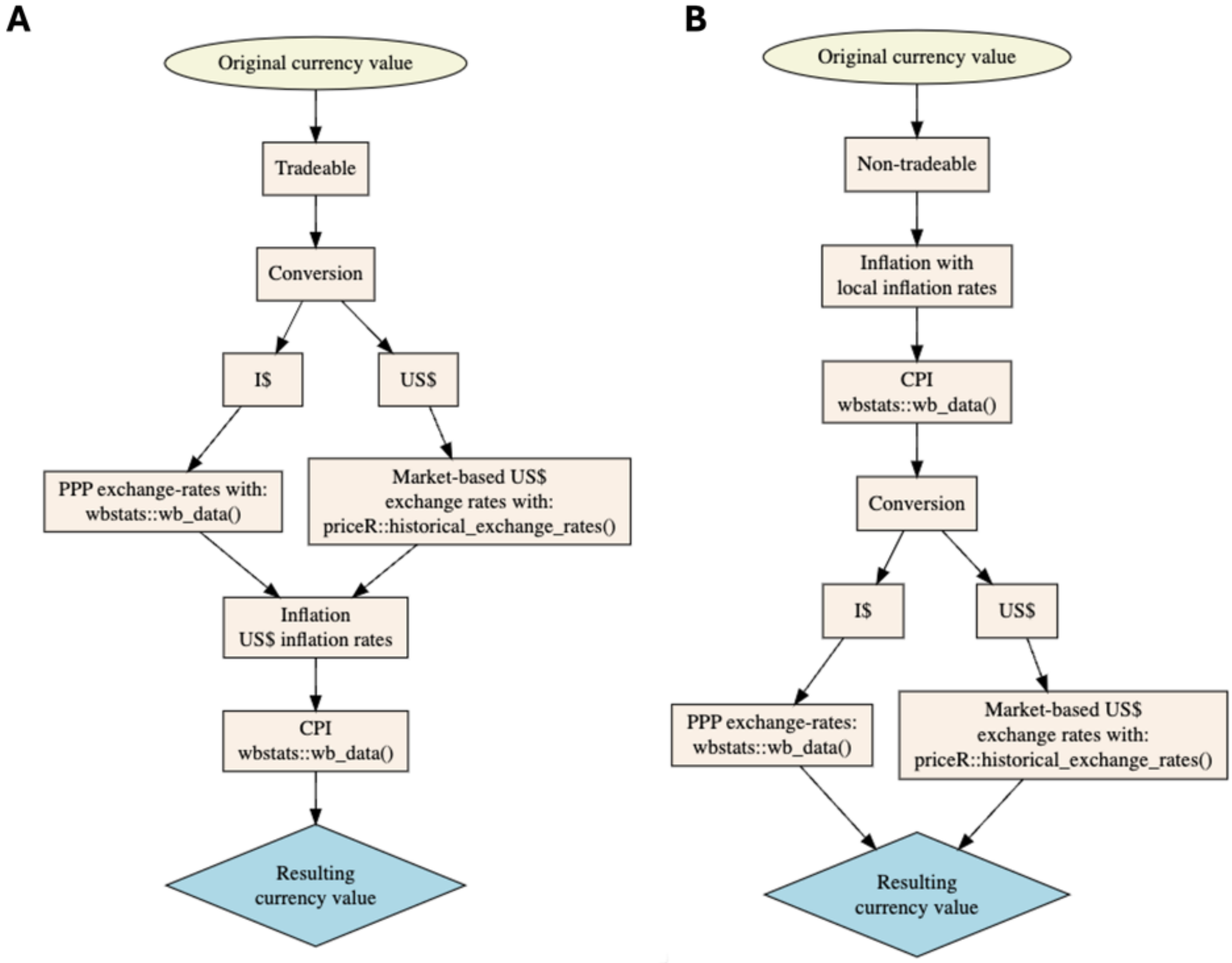

SUPPLEMENTAL DIGITAL CONTENT 5. Statistical analysis and ethical considerations

**Statistical analysis**	Categorical clinical variables are presented as counts (percentages) and analysed using the chi-Square or Fisher's exact test. Continuous variables are presented as mean (± standard deviation (SD)) or median (interquartile range (IQR)) depending on distribution. Group differences were tested with analysis of variance (ANOVA), Kruskal-Wallis, Student's t test, or Mann-Whitney U test, as appropriate. Logistic regression identified associations between the four selected variables (i.e. age, underlying condition, underweight, or clinical manifestation) and a composite outcome of mortality and/or disability. Statistical analyses were performed with SPSS version 28 (Armonk, USA). A P value <0.05 was considered statistically significant.
**Ethical considerations**	This study adhered to the Declaration of Helsinki and Good Clinical Practice guidelines. It was approved by Suriname's ethical review board (Ministry of Health reference: CMWO 17/'23).

SUPPLEMENTAL DIGITAL CONTENT 6. Mean annual incidence and 95% confidence intervals of IPD in children 0-15 years and children under 5 years of age in Suriname

	Children 0-15 years			<5 years		
	**IPD cases (n)**	**Population**	**Incidence[a] (95% CI)**	**IPD cases (n)**	**Population**	**Incidence[a] (95% CI)**
**2017**	7	152,900	4.6 (1.2-8.0)	7	53,800	13.0 (3.4-22.6)
**2018**	9	154,100	5.8 (2.0-9.7)	8	54,000	14.8 (4.5-25.1)
**2019**	8	155,300	5.2 (1.6-8.7)	7	54,400	12.9 (3.3-22.4)
**2020**	3	157,200	1.9 (0-4.1)	3	54,800	5.5 (0-11.7)
**2021**	8	158,300	5.1 (1.6-8.6)	8	55,000	14.5 (4.5-24.6)
**2022**	11[b]	158,300[c]	10.4 (5.4-15.5)	9[b]	55,000[c]	24.5 (11.5-37.6)
**Overall**	46	936,100	5.5 (4.0-7.0)	42	327,000	14.2 (10.1-18.3)

[a] Incidence per 100,000 children

[b] For 2022, data was available only up to August. The number of IPD cases was extrapolated to a full year (16.5 IPD cases for all children, 13.5 IPD cases for children under 5 years)

[c] Since 2022 population data has not been published, we used the 2021 population data as an estimate.

Abbreviations: CI = confidence interval; IPD = invasive pneumococcal disease

SUPPLEMENTAL DIGITAL CONTENT 7. Variables associated with mortality and/or disability in children with IPD

	**Univariable OR (95% CI)**	***P***	**Multivariable OR (95% CI)**	***P***
**Age**	1.0 (1.0-1.0)	0.48	1.0 (0.9-1.0)	0.20
**Underlying condition**	2.1 (0.6-7.6)	0.26	14.7 (0.7-296.5)	0.08
**Underweight**	0.6 (0.1-3.2)	0.52	0.1 (0.0-2.5)	0.15
**Clinical syndrome**				
Pneumonia	Reference	0.02	Reference	0.21
Meningitis	16.0 (2.7-96.5)	<0.01	8.7 (1.2-65.1)	0.04
Bacteremia/sepsis	2.0 (0.3-12.2)	0.45	3.1 (0.4-22.0)	0.26
Other	0.0 (0.0-..)	1.0	0.0 (0.0-..)	1.0

Abbreviations: CI = confidence interval; OR = odds ratio

SUPPLEMENTAL DIGITAL CONTENT 8. Mean (±SD) cost (US$) per patient per category

	**All patients (n=19)**	**No PICU admission (n=7)**	**PICU admission (n=12)**
Consultation	68.72 (±71.35)	25.58 (±19.39)	93.88 (±79.05)
Hospitalization	486.83 (±476.26)	209.87 (±226.38)	648.39 (±515.68)
Imaging	30.37 (±25.23)	22.13 (±12.06)	31.74 (±26.92)
Laboratory	56.97 (±51.75)	21.90 (±17.75)	71.58 (±54.67)
Consumables	50.72 (±61.10)	8.15 (±5.84)	75.55 (±65.31)
Medication	249.59 (±340.65)	44.06 (±84.48)	369.48 (±378.72)
Other	161.42 (±116.24)	0	161.42 (±116.24)
**Mean total cost per patient**	1,014.15 (±906.13)	309.63 (±338.09)	1,452.13 (±883.79)

SUPPLEMENTAL DIGITAL CONTENT 9. Mean (±SD) cost (I$) per patient per category

	**All patients (n=19)**	**No PICU admission (n=7)**	**PICU admission (n=12)**
Consultation	616.44 (±563.92)	209.14 (±126.78)	854.03 (±586.91)
Hospitalization	4,380.53 (±4,028.96)	1,646.64 (±1,604.84)	5,975.29 (±4,198.42)
Imaging	262.43 (±188.00)	164.38 (±89.71)	278.77 (±197.49)
Laboratory	542.05 (±507.12)	170.29 (±123.79)	696.95 (±528.68)
Consumables	147.38 (±177.56)	23.69 (±16.98)	219.54 (±189.78)
Medication	761.52 (±1,016.39)	145.38 (±246.50)	1,120.94 (±1,129.06)
Other	1,790.32 (±1,713.36)	0	1,790.32 (±1,713.36)
**Mean total cost per patient**	7,526.51 (±7,006.63)	2,193.45 (±2,032.09)	10,637.46 (±7,030.25)

SUPPLEMENTAL DIGITAL CONTENT 10. Characteristics of children with and without hospital bill available

	**All patients** (n=46)	**Hospital bill available (n=19)**	**Hospital bill not available (n=27)**	***P***
**Sex** (male n, %)	24 (52%)	10 (53%)	14 (52%)	ns
**Age** (months)	11 [5-28]	7 [5-28]	14 [6-29]	ns
**Underlying condition** (yes n, %)	17 (37%)	7 (37%)	10 (37%)	ns
**Underweight**[a] (yes n, %)	8 (19%)	2 (11%)	6 (25%)	ns
**Time between onset of symptoms and date of culture**[b] (days)	3 [2-3]	2 [2-3]	3 [2-4]	ns
**Clinical manifestation**				ns
Pneumonia	19 (41%)	10 (53%)	9 (33%)	
Meningitis	12 (26%)	6 (32%)	6 (22%)	
Bacteremia/sepsis	11 (24%)	2 (11%)	9 (33%)	
Other	4 (9%)	1 (5%)	3 (11%)	
**Site of infection**[c]				ns
Blood	39 (85%)	15 (79%)	24 (89%)	
Cerebrospinal fluid	9 (20%)	5 (26%)	4 (15%)	
Pleural aspirate	1 (2%)	1 (5%)	0 (0%)	
Abscess aspirate	4 (9%)	2 (11%)	2 (7%)	
Joint aspirate	1 (2%)	0 (0%)	1 (4%)	
**Laboratory tests**				
First Platelets[d] (x10e9/L)	402 [268-539]	476 [304-648]	353 [248-502]	0.04
First Leukocytes[e] (x10e9/L)	25 [18.4-34.4]	29.3 [19.9-35.4]	21.5 [10.9-27.6]	ns
First CRP[f] (mg/L)	220 [123-316]	240 [192-360]	191 [71-262]	ns
Maximum CRP[g] (mg/L)	243 [137-351]	311 [218-413]	197 [117-296]	ns
First Lactate[h] (mmol/L)	5.0 [2.1-13.4]	5.0 [2.7-14.1]	7.1 [1.7-.]	ns
**Outcome**				
PICU admission[i] (yes n, %)	18 (40%)	12 (63%)	6 (23%)	<0.01
PICU length of stay[j] (days)	13 [4-22]	16 [5-27]	6 [2-11]	ns
Hospital length of stay[k] (days)	9 [4-17]	11 [4-28]	9 [5-14]	ns
Mortality (yes n, %)	7 (15%)	4 (21%)	3 (11%)	ns
Disability[l] (yes n, %)	8 (40%)	5 (50%)	3 (30%)	ns

Values are reported as counts (percentages) or medians (interquartile ranges), unless stated otherwise
[a] Weight was known for 43/46 patients: 19/19 hospital bill available and 24/27 hospital bill not available.
[b] Time between onset of symptoms and date of culture was available for 29/46 patients: 13/19 hospital bill available and 16/27 hospital bill not available.
[c] Total count exceeds 46 because some patients had positive cultures from multiple sites.
[d] First Platelets data was available for 34/46 patients: 14/19 hospital bill available and 20/27 hospital bill not available.
[e] First Leukocytes data was available for 35/46 patients: 15/19 hospital bill available and 20/27 hospital bill not available.

[f] First CRP data was available for 33/46 patients: 15/19 hospital bill available and 18/27 hospital bill not available.

[g] Maximum CRP data was available for 31/46 patients: 14/19 hospital bill available and 17/27 hospital bill not available.

[h] First Lactate data was available for 10/46 patients: 8/19 hospital bill available and 2/27 hospital bill not available.

[i] PICU admission data was available for 45/46 patients: 19/19 hospital bill available and 26/27 hospital bill not available.

[j] PICU length of stay data was available for 16/18 PICU patients: 12/12 hospital bill available and 4/6 hospital bill not available

[k] Hospital length of stay data was available for 45/46 patients: 19/19 hospital bill available and 26/27 hospital bill not available.

[l] Disability data was available for 20/39 survivors: 10/15 hospital bill available and 10/24 hospital bill not available.
